# Effect of sodium alginate coating enriched with horsemint (*Mentha longifolia*) essential oil on the quality of bighead carp fillets during storage at 4°C

**DOI:** 10.1002/fsn3.202

**Published:** 2015-02-06

**Authors:** Ramin Heydari, Shahmir Bavandi, Seyed Roholla Javadian

**Affiliations:** Student of Fisheries, Qaemshahr Branch, Islamic Azad UniversityQaemshahr, Iran

**Keywords:** Alginate, bighead carp, edible coating, fish preservation, horsemint

## Abstract

Effect of sodium alginate coating enriched with horsemint essential oil (HEO) on the quality of bighead carp (*Aristichthys nobilis*) fillets at refrigeration temperature (4 ± 1°C) was studied. Bighead carp fillets were coated with neat sodium alginate (SA) and sodium alginate containing 0.5 and 1% v/v of HEO and their quality changes in terms of total volatile basic nitrogen (TVB-N), peroxide value (PV), thiobarbituric acid (TBA), and microbial counts were investigated. SA coating enriched with the essential oil could reduce the spoilage of the fillets and extend their shelf-life. Samples treated with SA-containing HEO showed significantly (*P* < 0.05) lower TVB-N content and lipid oxidation, as reflected by lower PV, FFA and TBA values during the storage period compared with the SA and control. The treatment also reduced the degree of microbial deterioration of the fillets (about 1.5 log_10_ CFU/g) more efficiently than the SA.

## Introduction

Fishery products are considered as rich source of high quality protein, essential vitamin and poly unsaturated fatty acids, and their consumption is on the rise all over the world. However, fresh fish products are usually more perishable than most other foodstuffs as a result of high water activity, neutral pH, relatively large quantities of free amino acids, presence of autolytic enzymes, and high percent of unsaturated fatty acids (Duan et al. 2010). This problem and the increasing request for high quality fresh seafood have intensified the search for technologies that favor fresh fish preservation. One of the most commonly employed methods for fish preservation is cold storage. Nevertheless, it does not sufficiently prohibit the quality deterioration of fish (Jeon et al. 2002). The quality of fresh fish products may be improved using novel technologies like modified atmosphere packaging (Fernández et al. 2009), edible coating (Fan et al. 2009; Song et al. 2011) and active bio-based packaging (Ojagh et al. 2010; Zhou et al. 2010).

Among these technologies, edible coatings can act as a barrier to the permeability of oxygen and water, thereby slowing oxidation reactions and retaining moisture. It is the main mechanism used by coatings to enhance quality and extend storage life (Gómez-Estaca et al. 2007). Among biopolymers used as coating, sodium alginate has gained special attention during the last years. Sodium alginate (SA) is a salt of alginic acid, a polymer of D-mannuronic and L-guluronic acid, which is extracted from brown seaweed and can be also synthesized from microorganisms (Olivas and Barbosa-Cánovas 2008; Vauchel et al. 2008). Its key properties, such as nontoxicity, biocompatibility, biodegradability, and reproducibility, have caused it to be used in many areas. These include food, pharmaceutical additives, biology or enzyme carrier, tissue engineering materials, and preparing the biodegradable or edible films such as alginate (Cho and Dreher 2001; Yang et al. 2009). However, it does not have antimicrobial or antioxidant properties when used as a coating while it can be a good carrier for natural bioactive compounds like essential oils.

Essential oils extracted from plants or spices are rich sources of biologically active compounds such as terpenoids and phenolic acids (Burt 2004). In general, essential oils, the odorous volatile products of an aromatic plant's secondary metabolism, are well-known as antimicrobial and antioxidant agents that could be used to control food spoilage, food-borne pathogenic bacteria and lipid oxidation (Kalemba and Kunicka 2003).

*Mentha longifolia* L. (common name: wild mint or horsemint) is considered a member of the large mint family, Lamiaceae. It is a fast-growing, perennial herb which can grow up to 1.5 m high in optimum conditions. This plant is an extremely variable species with a widespread distribution in Iraq, Mediterranean region, Europe and eastwards into Asia. It can be used in treating minor aches and sprains, and in nasal decongestants. Also, It is well known for its antipruritic, carminative, antiseptic, and stimulant properties (Al-Bayati 2009). Although some studies (Al-Bayati 2009; Ali Khan et al. 2011) have also reported good antibacterial properties for its extracts, their ability in a real-food system have not been studied. Moreover, in real food systems, the use of essential oils is often limited by the strong odor/taste they impart to foodstuffs. For this reason, the preservative effect of essential oils may be achieved by using low concentrations in combination with other preservation technologies (Mexis et al. 2009) like edible coating.

Thus, the present study was aimed to investigate the combined effect of sodium alginate coating and horsemint essential oil on the quality of bighead carp fillets during refrigerated storage. For achieving this aim, a number of chemical parameters, total viable count (TVC), and psychrotrophic viable count (PVC) were measured.

## Materials and Methods

### Materials

Sodium alginate (medium viscosity) and glycerol were obtained from Sigma-Aldreich Chemical Co., USA. The aerial parts of horsemint (*Mentha longifolia*) were purchased from a local market.

### Horsemint essential oil isolation

One-hundred grams of the dried aerial parts of horsemint (*M. longifolia*) was subjected to hydro-distillation for 4 h, using a Clevenger-type apparatus. The essential oil was isolated with a water-cooled oil receiver to decrease formation of artifacts due to overheating during hydro-distillation. The solvents were completely evaporated in an oven at 40°C. Isolation efficiency was 1% of the dried powder. The essential oils were collected and dried over anhydrous sodium sulphate and stored in a dark container at 4°C until used.

### Preparation of sodium alginate-based solutions

Alginate solution was prepared by dissolving 30 g of sodium alginate powder in 2 L distilled water to obtain a 1.5% w/v alginate solution using a magnetic stirring plate at 70°C and 1200 rpm for 30 min, then cooled to room temperature. Then Tween 80 (0.25 g/g of essential oil) was used as an emulsifier to help create a uniform and stable distribution in the alginate matrix, was added to the mixture and striied in 40°C for 30 min, the isolated horsemint essential oil (HEO) was incorporated into the prepared solution at several concentrations (0.5, and 1.0% w/v on the basis of sodium alginate solution). The final solution was homogenized with Ultra-Turrax (IKA T25-Digital Ultra-Turrax, Staufen, Germany) at 9000 rpm for 2 min. The resulting solution was degassed under vacuum for 30 min in order to remove all bubbles.

### Preparation of bighead carp fillets, coating and storage

Thirty-six live bighead carp (*Aristichthys nobilis*) with an average weight of 1000 ± 100 g were obtained from a local aquaculture farm. In 1 h, they were transported to the laboratory in sealed foamed polystyrene boxes containing flaked ice. Then, the fish were gutted, skinned, filleted, and washed with tap water in the laboratory. Fifteen fillets (100 ± 10 g) for each treatment were randomly subjected to one of four treatments as presented in the following:

C: control, without treatment
SA: coated with sodium alginate
SA-HEO-0.5%: coated with sodium alginate containing 0.5% HEO
SA-HEO-1%: coated with sodium alginate containing 1% HEO

The fish fillets were dipped for 30 sec in 500 mL of the each coating solution. Then, the coated fillets stood for 2 min, followed by a second immersion in CaCl_2_ (Sigma-Aldrich Chemical Co.) for 30 sec to achieve better crosslinking (Lu et al. 2009). Next, the samples were allowed to drain completely in ambient condition for about 30 min. Finally, they were stored at 4 ± 1°C until testing. Chemical and microbiological analyses were performed at 4-day intervals to determine the overall quality of the fish for 16 days.

### Chemical analysis

#### Proximate composition

The moisture content and crude ash were determined in an oven at 103 and 550°C, respectively, until the weight became constant. The total crude protein was determined by Kjeldahl's method (AOAC 2002) and the lipid content was analyzed according to the procedure of Bligh and Dyer (1959). All measurements were repeated three times for studying repeatability.

#### The total volatile basic nitrogen (TVB-N)

TVB-N of the bighead carp samples was measured by the micro-diffusion method as described by Goulas and Kontominas (2005). The values were reported in mg N/100 g of fish. Measurements were repeated three times for studying repeatability.

#### Evaluation of lipid oxidation

The peroxide value (PV) was expressed in mEq oxygen/lipid and determined in the total lipid extracts according to the method of Pearson (Egan et al. 1997). Free fatty acid (FFA) was determined by the procedure explained by AOAC (2002) and its content was expressed as percentage of oleic acid. The colorimetric method described by Kirk and Sawyer (1991) was used to measure the thiobarbituric acid (TBA) value in fish fillet for secondary lipid oxidation products evaluation. All measurements were repeated three times for studying repeatability.

### Microbiological analysis

The pour plate method was used to determine total viable count (TVC) and total psychrotrophic count (TPC). Ten grams of the fish minced sample was aseptically taken and homogenized in 90 mL of sterile 85% NaCl solution with a blender (HBM-400B, HBM Biomed, Tianjin, China) at room temperature. Appropriate dilutions were serially prepared and then 1 mL of each was spread onto plate count agar media (Merck, Darmstadt, Germany). The prepared plates were incubated at 37°C for 2 days for TVC, and at 10°C for 7 days for TPC. All counts were expressed as log colony-forming units (CFU)/g and performed in triplicate.

### Statistical analysis

The differences among all measurements were evaluated by one-way analysis of variance (ANOVA). Duncan's multiple range tests were used to compare the means to identify which groups were significantly different from other groups. Significance was defined at *P* < 0.05. All data are presented as mean ± SD.

## Results and Discussion

### Chemical changes

The composition of fish can affect the sensory properties that greatly influence the acceptability of fish as food. It may also affect microbial growth in fish products (Sallam 2007). The proximate composition of bighead carp fillets is summarized in Table1. Fish samples contained a low amount of fat (2.21%) and 18.79% of protein which was reported by Abdollahi et al. (2014) for silver carp fillets. Nevertheless, fish body composition changes greatly from one species to another and one individual to another. Thus, notable changes can be seen in the components of fish muscle (Pacheco-Aguilar et al. 2000).

**Table 1:** Proximate composition of bighead carp fillets (C: control, without treatment, SA: coated with sodium alginate, SA-HEO-0.5%: coated with sodium alginate containing 0.5% HEO, SA-HEO-1%: coated with sodium alginate containing 1% HEO)

Protein (%)	Fat (%)	Moisture (%)	Ash (%)
18.79 ± 0.34	2.21 ± 0.22	77.92 ± 0.43	0.09 ± 0.08

**Figure 1:**
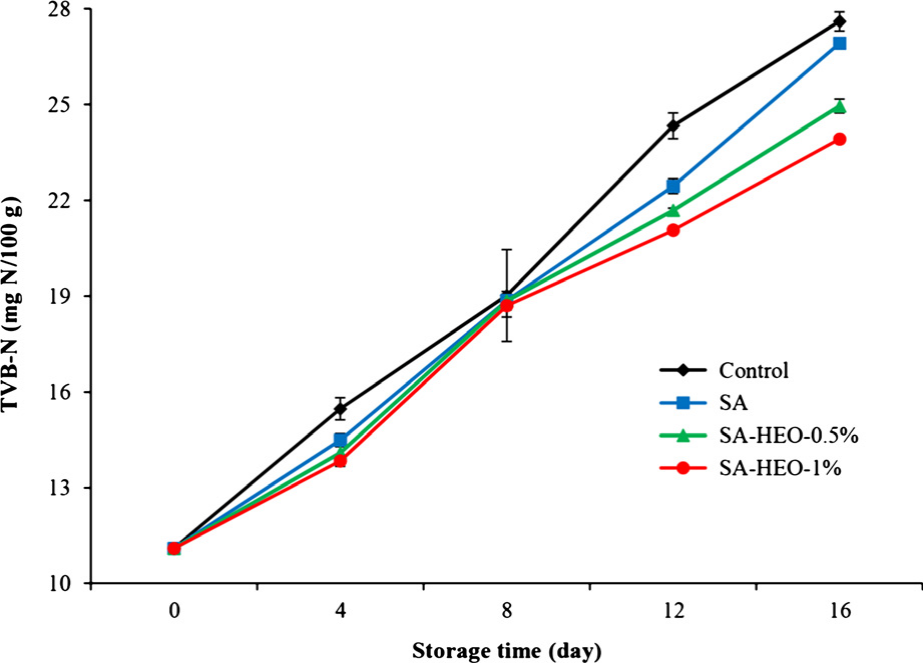
Changes in the total volatile basic nitrogen (TVB-N) of fish fillets during refrigerated storage (C: control, without treatment, SA: coated with sodium alginate, SA-HEO-0.5%: coated with sodium alginate containing 0.5% HEO, SA-HEO-1%: coated with sodium alginate containing 1% HEO).

TVB-N is widely studied as an indicator of deterioration of fish muscle and measures the compounds composed of ammonia and primary, secondary, and tertiary amines (Fan et al. 2009; Abdollahi et al. 2014). According to Leroi et al. (1998), fish flesh with a level of 30 mg TVB-N per 100 g is usually regarded as spoiled. The TVB-N values of bighead carp fillets are summarized in Fig.1. The initial TVB-N value of the fillets was 11.10 mg/100 g which showed the good quality of the fresh samples in that, freshwater fish muscle has 10–20 mg/100 g TVB-N after harvesting (Alçiçek 2011). The value of TVB-N increased progressively with the time of storage for all fish samples. This observed increase during storage may be related to several enzymatic processes, including the deamination of free amino acids, degradation of nucleotides, and oxidation of amines or microbial activities (Lu et al. 2009; Alçiçek 2011). However, TVB-N content of the samples coated with sodium alginate was significantly lower than the control at the last 8 days of the storage period (*P* < 0.05). This results was in agreement with previous observations about refrigerated northern snakehead (Lu et al. 2009), bream (Song et al. 2011), and trout fillets (Hamzeh and Rezaei 2012) coated with sodium alginate. This may be explained by reducing water loss or acting as a barrier for oxygen in coated fillets. In addition, TVB-N content of the samples coated with sodium alginate containing horsemint essential oil was significantly lower than the control and SA treatments during the storage period (*P* < 0.05). This may be related to either a more rapidly reduced bacterial population or decreased capacity of bacteria for oxidative deamination of non-protein nitrogen compounds or both caused by antimicrobial properties of HEO (Song et al. 2011). Similarly, other authors reported lower TVB-N content in fish fillets treated with other plant essential oils like cinnamon essential oil (Ojagh et al. 2010) and thyme essential oil (Alçiçek 2011).

### Lipid oxidation

The peroxide value (PV) provides a measure of the hydroperoxides which are the primary products of auto-oxidation and are odorless. Nevertheless, their decay leads to the formation of a wide range of carbonyl compounds, hydrocarbons, furans, and other products that contribute to the rancid taste of decaying food (Yanishlieva and Marinova 2001). Table2 shows changes in PV of bighead carp fillets during the storage period. Initial values of PV were very low (0.25 meq O_2_/kg on average) in the fresh fillets. As can be seen, the PV of the fillets increased gradually in all treatments during the period. During refrigeration storage, values were generally much greater for control at all sampling points, while any significant difference was not found up to days 8. Thereafter, significant differences (*P* < 0.05) were observed between samples with HEO and the C or SA. These results may be attributed to the antioxidant activity of HEO which is related to its polyphenol contents. As explained by Turhan et al. (2009), phenolic antioxidants do not act as oxygen absorbers; rather, they inhibit the formation of fatty acid free radicals, which do react with or absorb oxygen in the auto-oxidation process. This performance delays the onset of the auto-oxidative process in fat or oil (Abdollahi et al. 2014). Ojagh et al. (2010) also reported lower amounts of PV in trout fillets coated with chitosan containing cinnamon essential oil.

**Table 2:** Changes in peroxide value (PV), free fatty acid (FFA), and thiobarbituric acid (TBA) value of coated fillets during storage (C: control, without treatment, SA: coated with sodium alginate, SA-HEO-0.5%: coated with sodium alginate containing 0.5% HEO, SA-HEO-1%: coated with sodium alginate containing 1% HEO)

Attributes	Treatment	Storage period (days)				
		0	4	8	12	16
PV	C	0.25 ± 0.05^a^	2.01 ± 0.08^a^	3.43 ± 0.25^a^	4.50 ± 0.25^a^	5.79 ± 0.05^a^
(mEq oxygen/kg oil)	SA	0.25 ± 0.05^a^	1.87 ± 0.07^a^	2.85 ± 0.40^ab^	4.21 ± 0.20^a^	5.19 ± 0.15^b^
	SA-HEO-0.5%	0.25 ± 0.05^a^	1.90 ± 0.11^a^	2.45 ± 0.291^b^	3.38 ± 0.24^b^	4.03 ± 0.01^c^
	SA-HEO-1%	0.25 ± 0.05^a^	1.81 ± 0.09^a^	2.36 ± 0.51^b^	3.30 ± 0.60^b^	3.93 ± 0.15^c^
FFA (% oleic acid)	C	0.20 ± 0.02^a^	1.96 ± 0.03^a^	3.07 ± 0.09^a^	5.48 ± 0.14^a^	6.01 ± 0.14^a^
	SA	0.20 ± 0.02^a^	1.90 ± 0.02^a^	2.97 ± 0.17^a^	4.14 ± 0.03^b^	5.09 ± 0.08^b^
	SA-HEO-0.5%	0.20 ± 0.02^a^	1.87 ± 0.25^a^	2.12 ± 0.03^b^	3.39 ± 0.09^c^	4.33 ± 0.09^c^
	SA-HEO-1%	0.20 ± 0.02^a^	1.81 ± 0.32^b^	2.22 ± 0.09^b^	2.84 ± 0.17^d^	3.92 ± 0.14^d^
TBA (mg MDA/kg)	C	0.10 ± 0.02^a^	0.42 ± 0.05^a^	1.95 ± 0.13^a^	3.51 ± 0.13^a^	3.17 ± 0.08^a^
	SA	0.10 ± 0.02^a^	0.40 ± 0.04^ab^	1.67 ± 0.24^a^	3.12 ± 0.24^b^	2.56 ± 0.23^b^
	SA-HEO-0.5%	0.10 ± 0.02^a^	0.33 ± 0.07^ab^	1.19 ± 0.08^b^	2.19 ± 0.08^c^	1.94 ± 0.13^c^
	SA-HEO-1%	0.10 ± 0.02^a^	0.29 ± 0.04^b^	0.96 ± 0.60^b^	1.96 ± 0.60^c^	1.76 ± 0.30^c^

^a,b,c^Different small letters in the same column, represents significant difference (*P *<* *0.05).

The progress of lipid hydrolysis was studied by measuring free fatty acids (FFA) which are triacylglycerols products formed either by chemical- or enzyme-mediated hydrolysis (Barthet et al. 2008). Table2 shows change in FFA acid contents of bighead carp fillets during 16 days of storage. The FFA content of all treatment samples increased from an initial amount of 0.20 (% oleic acid) in the fresh samples, until it reached a maximum (C = 6.01, SA = 5.09, SA-HEO-0.5% = 4.33, and SA-HEO-1% = 3.92%) on the day 16. The overall increase displays hydrolytic oxidation in the fillets caused by internal or bacterial enzymes and the decrease may be related to the interaction of triacylglycerols products with proteins (Pereira De Abreu et al. 2011). In the final stages of refrigerated storage, the samples of SA-HEO-0.5% and SA-HEO-1% showed significantly (*P* < 0.05) lower content of FFA in comparison with C and SA (about 33 and 25%, respectively). These results coincide with those reported by Ozogul et al. (2010) for sardines fillets treated with rosemary extract. The lower content of FFA in the samples treated with HEO may be due to the influence of the essential oil on meat enzymes and their activity (Silva and Ammerman 1993).

Second stage auto-oxidation during chilled storage of bighead carp fillets was measured by variation of thiobarbituric acid (TBA) values. Changes in the TBA values of different treatment groups during the storage period are summarized in Table2. As can be seen, the initial value of TBA was around 0.10 mg MDA/kg, closing to the value reported for silver carp by Fan et al. (2009). The TBA value of the bighead carp samples increased through the whole storage period, especially in the control samples which shows secondary lipid oxidation in the samples. Samples coated with sodium alginate showed significantly (*P* < 0.05) lower amount of TBA compared with those of the control by the 12th day until the end of the period. Similarly, Souza et al. (2010) also reported lower TBA values in salmon fillets coated with chitosan by the 12th day. Oxygen barrier properties of alginate may have contributed to the control of lipid oxidation in bighead carp fillets. However, Lu et al. (2009) did not find lower TBA value for northern snakehead (*Channa argus*) fillets coated with alginate-calcium. Moreover, the TBA value in fillets coated with sodium alginate enriched with HEO was lower than that in control and alginate coated fillets which may be related to the antioxidant properties of essential oil. It has been suggested that phenolic compounds are able to donate a hydrogen atom to the free radicals thus stopping the propagation chain reaction during lipid oxidation process (Singh et al. 2006).

### Microbiological changes

**Figure 2:**
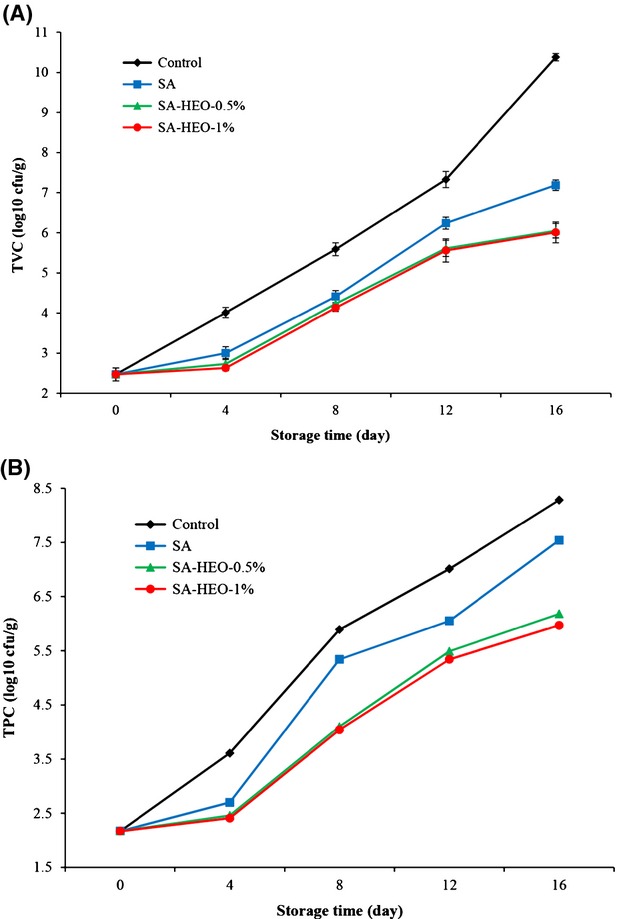
Changes in (A) total viable count (TVC) and (B) total psychrotrophic count (TPC) of fish fillets during refrigerated storage. (C: control, without treatment, SA: coated with sodium alginate, SA-HEO-0.5%: coated with sodium alginate containing 0.5% HEO, SA-HEO-1%: coated with sodium alginate containing 1% HEO).

The composition of fish muscle makes it favorable for microbial growth. Thus, fish spoiling occurs during storage mainly as a result of microbial activity (Souza et al. 2010). Changes in total viable count (TVC) and total psycrotrophic count (TPC) of bighead carp fillets are shown in Fig.2A and B. A low bacterial count (2.44 and 2.17 log_10_ CUF/g) was initially observed in fresh bighead carp fillets. It shows the high quality of samples used in the present study (ICMSF 1986). As can be seen, the TVC and TPC of all samples increased with storage time but the value increased faster for control. The TVC and TPC of control increased quickly and exceeded the maximum acceptable limit of 6 log_10_ CFU/g (ICMSF 1986) for freshwater and marine fish at day 8th. TVC of fillets coated with sodium alginate increased slower than the control and exceeded the limit at the 12th. The same trend was also observed about TPC. The significant reduction in TVC and TPC observed in the bighead carp fillets coated with SA may be due to fact that the coating acts as a barrier against oxygen transfer and leads to inhibition of growth of the aerobic bacteria (Song et al. 2011). These results agreed with those reported by Lu et al. (2009) and Song et al. (2011) for refrigerated northern snakehead and bream, respectively, coated with sodium alginate. The TVC of SA-HEO-0.5% and SA-HEO-0.5% did not exceed the limit value during the entire storage and no difference was observed between them (*P* > 0.05); they reached 6.05 and 6.01 log_10_ CFU/g, respectively, on day 16. The antimicrobial activity of horsemint extract and essential oils had been reported by Ali Khan et al. (2011) and Al-Bayati (2009). These results coincide with those reported by Lu et al. 2010 and Ojagh et al. (2010) for refrigerated northern snakehead and rainbow trout fillets coated with alginate-calcium and chitosan enriched with cinnamon essential oil, respectively.

## Conclusions

The combined effect of sodium alginate coating and horsemint essential oil on the quality of refrigerated bighead carp fillets was studied. Sodium alginate coating enriched with the essential oil could reduce the spoilage of the fillets and extend their shelf-life. Samples treated with sodium alginate containing horsemint essential oil showed significantly lower TVB-N content and lipid oxidation, as reflected by lower PV, FFA, and TBA values during the storage period compared with the SA and control. The treatment also reduced the degree of microbial deterioration of the fillets more efficiently than the sodium alginate. Antioxidant and antibacterial effects of sodium alginate coating and horsemint were more pronounced when a horsemint was used at 1% concentration. Therefore, these coatings could be promising alternatives to synthetic materials in food formulation, possibly contributing to improve food quality and prolong shelf life of fresh produce.

## Conflict of Interest

None declared.
